# The Local Application of Cold in Acute Pneumonia

**Published:** 1896-01

**Authors:** Thomas J. Mays

**Affiliations:** Philadelphia, Pa.


### THE LOCAL APPLICATION OF COLD IN ACUTE PNEUMONIA.

By THOMAS J. MAYS, A.M., M.D.,
PHILADELPHIA, PA.

In the *Medical News* of June 24, 1894, I presented a collection of fifty cases of acute pneumonia which were treated with ice-cold applications to the chest. Since that time, and especially during the early part of the present year, when through the kindness of the medical press I was enabled to extend a general invitation to the profession for reports of pneumonia cases which were similarly treated, I have received 145 additional reports—making a total of 195 cases.

Of the 145 cases which are comprised in the present collection only 93 are reported with sufficient detail to enable me to tabulate them; close notes of the remainder were either not taken, or were lost after they had been obtained by the observers.

*Sex.* Among the total number the sex is noted in 143 cases, of which 67 are males and 76 females.

*Age.* The age is given in 143 cases. The number of cases occurring in each decade is as follows: Below one year (three weeks, six and a half months, and eight months, respectively), 3; between one and ten years, 27; ten and twenty, 34; twenty and thirty, 26; thirty and forty, 29; forty and fifty, 16; fifty and sixty, 2; sixty and seventy, 5; seventy and seventy-five, 1.

*Highest range of temperature.* This is recorded in 126 cases—48 males and 78 females. The highest temperatures attained in these cases were as follows: In two cases it was 100°; in two, 101°; in three, 102°; in five, 102½°; in nineteen, 103°; in ten, 103½°; in thirty-seven, 104°; in ten, 104½°; in twenty-one, 105°; in six, 105½°; in six, 106°; in one, 106½°; and in four, 107° F.

*Days of crises or subsidence of fever.* These are noted in 113 cases, and they occurred as follows: In 8 cases at the end of the first day; in 18 at the end of the second day; in 7 at the end of the third day; in 18 at the end of the fourth day; in 16 at the end of the fifth day; in 13 at the end of the sixth day; in 11 at the end of the seventh day; in 6 at the end of the eighth day; in 9 at the end of the ninth day; in 1 at the end of the tenth day; in 3 at the end of the eleventh day; and in three at the end of the twelfth day.

*Number of cases of single and double pneumonia.* There were 108 cases of single and 31 cases of double pneumonia, or nearly four times as many of the former as of the latter. The average highest temperature of the single pneumonia cases was 104.51°, while that of the double cases was 104.62°. The percentage of deaths among the former was 3.70 and among the latter 6.45.

*The Mortality.* Among the 195 cases there were 7 deaths, 4 males and 3 females, or a mortality rate of *3.58 per cent.* The oldest of these was seventy-four and the youngest was sixteen years. Three were double and four were single cases of pneumonia. The highest temperature attained among these cases was 104° and the lowest 102°, or an average of $103\frac{2}{3}$°, there being only an average difference of 0.34° between those who died and those who recovered. The personal history of those who died was as follows:

1. Adult, had a chill ten days before he was seen, and died three days after being admitted. When he came to the hospital he was cyanotic, delirious, and had excessive diarrhea. His pneumonia was double.

2. Aged thirty-seven years, had diarrhea and delirium tremens, and his condition was complicated with chronic lead-poisoning. His highest temperature was 102°.

3. Aged twenty-eight years, an acute exacerbation of chronic pneumonia.

4. Aged twenty-six years, double pneumonia; was admitted four days after the chill. His temperature was 103° and respiration 45; breathing very labored and painful. Died sixteen hours after admission, of apnea. *Post-mortem* examination showed chronic pthisis in both apices, a cavity, with chronic consolidation around it, in left apex. The lower lobes of both lungs were hepatized, and the middle lobe of right side was in a state of congestion.

5. Sixteen years old; admitted four days after chill with double pneumonia, at which time she had an offensive diarrhea, bloody stools, and hemoptysis, and was cyanosed and in a stupor. Died on the evening of the second day after admission.

6. Aged seventy-four years. Highest temperature 102°. Died on the eighth day. Ice-bags were applied from the first to the third day.

7. Aged forty years, whole of right lung involved. Highest temperature 194°. Ice applied on first and second days. Died on the third day.

*Comparison of Results.* Whatever opinion we may hold in regard to the value of any treatment, it is quite obvious that in the long run the verdict will favor that one which shows the smallest mortality rate. I am well aware that statistics are often unreliable unless they roll up into large figures, yet I believe that the number of cases which I present for consideration will form a basis on which to rest an opinion of the treatment here advocated; but before drawing any positive conclusions I will briefly inquire into the results which have been obtained by various other forms of treatment of pneumonia. Dr. Osler reports that out of 1,012 cases treated in the Montreal General Hospital 20 per cent. died, and that in the Charity Hospital of New Orleans the mortality rate was 20.01 per cent.; of 1,000 cases of pneumonia treated in the Massachusetts General Hospital from 1822 to 1889 the death rate was 25 per cent. Dr. Hartshorne states that the mortality rate from the disease in the Pennsylvania Hospital during the years 1884, 1885, and 1886 was a little more than 31 per cent. Louis treated 107 cases of which 32, or about 30 per cent., died. Grisolle had a mortality of 16 per cent. in 232 uncomplicated cases. Rasori treated 648 cases of pneumonia with large doses of tartrate of antimony, of which about 22 per cent. died. With the same medicine, Grisolle lost 18.8 per cent. of 154 cases, and Dietl 20.7 per cent. of 106 cases. During a period of sixteen years previous to 1861 Dr. Huss, of Stockholm, treated 2,616 cases, with a death rate of 10.74 per cent. Of 129 cases treated on the restorative plan by Dr. Bennett, of Edinburgh, only four died, giving a mortality of 3.1 per cent. Dr. Hegele, head physician of the Wurzburg Hospital, during 1848 and 1849, treated 40 cases with cold water, without a single death.

Now what conclusions are we to draw from a death-rate which varies all the way from 30 per cent. to nothing? Are we to believe that all pneumonics recover under certain methods of treatment, and that a large proportion of them will necessarily die under others? No one would be justified in making quite such a reckless assertion, yet it must be admitted that the truth lies somewhere between these two extremes. But in order to get at something more tangible, let us first find the rate of natural recovery from this disease. What proportion get well under no treatment whatever, and what proportion die under such circumstances? After having ascertained this we will be in a better position to estimate the worth or worthlessness of any treatment. It is very fortunate that this important link has been furnished. In 1844, '45, and '46 Dr. Dietl, a Viennese physician, treated 189 cases of pneumonia practically without medicine, and with a death-rate of 7.4 per cent. From 1847 to 1850 he treated a second series of 750 cases, with a death-rate of 9.2 per cent. That this represents, at least approximately, the rate of natural recovery from pneumonia is also confirmed by the results of pure homœopathic practice, which may be regarded as equivalent to the let-alone or do-nothing treatment. Thus, out of 94 cases of pneumonia treated in the homœopathic section of Leopoldstadt Hospital in Vienna, 9.57 per cent. died; out of 24 cases of pneumonia treated in the same institution by Drs. Wurmb and Caspar, 12.55 per cent. died; and Dr. Tessier, of Paris, treated 41 cases of pneumonia, homœopathically, with a mortality of 7.3 per cent.

If these figures are reliable, and there is no evidence that they are not, they show that, as a rule, the natural tendency to recovery in pneumonia is about 90 per cent., and that, therefore, any treatment which is not capable of holding the death-rate down to or below 10 per cent. is not only worthless, but actually mischievous. So far as I apprehend this, however, it only applies to an ordinary aggregate of favorable and unfavorable cases of pneumonia, and not to exceptional instances like those which are met in fatal epidemics of pneumonia, or in a large number of successive cases of alcoholic, senile, or latent pneumonia, as may happen in the experience of any medical man with a large public or private practice.

When we come to compare the results of the ice-cold treatment of pneumonia with those which have been obtained from other forms of treatment, it is safe to say that the former are infinitely more satisfactory than the latter. Although Dr. Bennett's figures excel those which are presented in my collection by about one-half of one per cent., it must not be forgotten that his results are entirely overshadowed by Dr. Hegelé's 40 cases, which were treated hydropathically without a single death. Moreover, it must not be overlooked that, so far as my knowledge extends, Dr. Bennett's observations have not been duplicated by anyone on the lines laid down by him; while, on the other hand, this is not the case with the cold treatment; its results in my collection have not been secured by a single individual only, but by as many as thirty-four different observers, among whom are a number who have seen a score of cases, and many more a lesser number in succession, without a single death. This of itself speaks volumes in favor of the method here advocated, for it shows that the personal equation of the practitioner cannot enter very largely into the success of the treatment.

Then, again, I express the hope that these statistics will tend to put to rest the mischievous and irrational dogmatism which regards pneumonia as a self-limited disease, like smallpox and scarlatina, and consequently wholly beyond the control and influence of medicine. Not only is this position refuted by the fact that the death-rate in pneumonia when treated by local cold applications is lower than if the disease is allowed to go untreated, but its unsoundness becomes still more apparent when we learn that the cold treatment hastens the appearance of the day of crisis.

Now if these figures have any value, they indicate that cold *has* a marked and decided influence on the pneumonic process, not only in bringing it to a favorable termination, but to materially shorten its course. This abortive power of the ice has been noticed and spoken of by many of the observers in my collection, and I believe that it demonstrates the great value of the remedy more than anything else.

*Mode of death in pneumonia, and its therapeutic indications.*—Now a few words in regard to the tendency toward death in pneumonia. A high fever, high pulse-rate, frequent and difficult respiration, extensive exudation, etc., are universally regarded as critical and serious symptoms and conditions of this disease. On deeper examination I believe, however, that these are mere superficial manifestations of grave disorder of the nervous system below, and that the gravity and intensity of each attack are universally related to the degree of resistance which is offered by the nervous system against the ingress of the disease. This is corroborated by the common observation that pneumonia is ushered in by intense nervousness, violent headache, active delirium, local muscular spasm, and general convulsions. Lemaire reports a case (*Centralbl. f. Nervenheilkunde*, 1888, vol. ii., p. 680) in which two attacks of pneumonia were preceded each time in the same individual, the first at the age of thirty-two, and the second at forty, by genuine epileptic seizures, and this in a person who had not been subject, before or afterward, to the latter disease. The close affinity between pneumonia and diseases of the nervous system is also shown in the almost constant association of the former affection with cebro-spinal meningitis, and with other acute diseases of the brain and the upper portion of the spinal cord. That disorder of the nervous system is capable of originating pneumonia is by no means a new doctrine. A few years ago Dr. Hughlings Jackson, in discussing a paper on "Pulmonary Paresis," read by Dr. B. W. Richardson before the London Medical Society, said that he regarded acute pneumonia as a form of herpes zoster of the pneumogastric nerves. Dr. Fernet, in a paper on "Neuritis of the Pneumogastric Nerves as a Cause of Acute Pneumonia," expresses the conviction that the so-called fibrinous pneumonia is a herpes of the lungs brought about by disease of the pneumogastric nerves. Professor Baelz, in the course of his lectures in the Tokio University of Japan, teaches that pneumonia is a reflex vasomotor exudation neurosis. It is thus seen that this line of thought has occupied some of the foremost men in the medical profession.

From this we learn that one of the most threatening dangers in pneumonia comes from a defective supply of nerve force to the lungs. This is indicated, not so much by a great frequency of breathing, as it is by a frequent and laborious respiration. The patient who lies there moaning with and struggling for each breath, and who complains of pain and distress in the gastric region—the evidence of an overworked diaphram—must alawys be regarded with the gravest concern. Here the tendency toward death is pulmonary exhaustion—a condition sometimes found without very marked extension of the exudation process or much fever, and most frequently in the aged or in those whose nervous systems are overworked or exhausted by alcoholic excess, etc. Equal to the danger of the above condition is the extension of the disease in the lungs. When this covers a large area, as in double pneumonia, there is marked interference with the function of breathing, and the patient tends to die for the want of sufficient aërating surface.

The next in importance are hyperpyrexia and a weak heart. It is not necessary to say anything about the detrimental effects of high fever in pneumonia. It is also well recognized that, in virtue of its close affiliation with the lungs, a weak and disordered heart is a constant accompaniment of pneumonia.

The therapeutic indications in the management of pneumonia are, therefore, as follows: (1) Suppression and limitation of the process of exudation; (2) Support of the nervous system and particularly of the pulmonary nerve supply; (3) Reduction of fever; and (4) Maintenance of the function of the heart.

Now what part does cold play in filling these indications? It cannot fill them all, but it covers those of greatest importance. First of all it reduces the pyrexia, strengthens the pulse, tones up the heart, diminishes the pain in the chest, alleviates difficulty of breathing, and gives greater general comfort to the patient. It is capable, however, of doing a great deal more. In virtue of its power to stimulate nerve function and to contract small blood vessels it promotes the pulmonary circulation, relieves stasis, hastens resolution, and disperses the products of exudation. Strychnine in large doses to sustain respiratory and cardiac innervation; concentrated food of the most nourishing character, like fresh beef-juice, milk, brandy, etc., to support the constitution; digitalis to maintain the heart; oxygen by inhalation to relieve the breathing; morphine to produce sleep; ice-cap to the head to diminish restlessness; and strapping of the chest in case of great pleuritic pain, are some of the agents which will fill most of the remaining indications.

